# Unveiling the longitudinal reciprocal relationship between burnout and engagement among adolescent athletes in sport schools

**DOI:** 10.1002/jad.12426

**Published:** 2024-10-14

**Authors:** Joni Kuokkanen, Milla Saarinen, Daniel J. Phipps, Johan Korhonen, Jan‐Erik Romar

**Affiliations:** ^1^ Faculty of Education and Welfare Studies Åbo Akademi University Turku Finland; ^2^ The Child and Youth Sport Research Centre Norwegian School of Sport Sciences Oslo Norway; ^3^ Faculty of Sport and Health Sciences University of Jyväskylä Jyväskylä Finland; ^4^ School of Applied Psychology Griffith University Mt Gravatt Australia

**Keywords:** adolescent athletes, burnout, demands–resources model, dual career, engagement, lower secondary sport schools

## Abstract

**Introduction:**

Burnout and engagement are pivotal for adolescents' well‐being and have received extensive attention in the educational literature. However, less is known about how these factors develop and interact within and between school and sport when adolescent athletes follow dual (school and sport) careers. The aim of this study was to examine the reciprocal relationship between engagement and burnout in school and sport through a cross‐lagged analysis of longitudinal associations.

**Methods:**

A sample of 165 Finnish adolescent athletes (aged 14.5 years, 48.9% girls) enrolled in lower secondary sport schools (i.e., junior high schools) participated in a survey in spring 2019 (i.e., in Grade 8) and 1 year later, in spring 2020 (i.e., in Grade 9).

**Results:**

Engagement and burnout had significant autoregressive relationships within school and sport. Furthermore, low levels of sport engagement in Grade 8 predicted sport burnout in Grade 9, and low levels of school burnout in Grade 8 predicted school engagement in Grade 9. Regarding cross‐domain effects, high levels of school engagement in Grade 8 predicted low levels of sport engagement in Grade 9.

**Conclusions:**

The study shows that school and sport are distinct domains with unique associations between engagement and burnout in each domain. It also shows that adolescent athletes may maintain high school engagement by reducing their sport engagement. These findings highlight the need for sport school practitioners to develop intervention programs to address adolescents' school and sport needs and to support their holistic well‐being.

## 1 INTRODUCTION

Roughly half of 13–15‐year‐old European adolescents and 60% of US adolescents participate actively in sports (Blomqvist et al., 2023; Sports & Fitness Industry Association, 2020). Sport participation patterns vary across contexts, with various systems providing opportunities for sport participation (Lim et al., 2011). Unlike American students, who benefit from well‐organized school sport systems, European adolescents typically pursue their school studies while training and competing in sports through sport clubs (Kelley et al., 2018). Consequently, many European countries are developing sport schools alongside sport clubs to facilitate effective combinations of school and sport. Adolescent athletes may pursue a dual career (DC) by combining their athletic pursuits with studies in sport schools (Morris et al., 2021).

This study was conducted in Finland, where students start school at age 7 and complete 9 years of compulsory education by age 15 or 16, including primary school (Grades 1–6, similar to elementary school in the United States) and lower secondary education (Grades 7–9, comparable to junior high school in the United States). After completing compulsory education, students must choose between a general academic track or a vocational track in upper secondary education (age 16–18, similar to the later grades of high school in the United States). DC athletes can also apply for specialized upper secondary sport schools, which provide tailored support for combining education with high‐performance sports. Due to the increasing demands of school and sports during lower secondary school, adolescents often choose to drop out of sports (Blomqvist et al., 2023). Thus, there is a crucial need to identify the factors that influence the well‐being of adolescent athletes aged 13–15 years.

Burnout and engagement are central factors influencing adolescent athletes' well‐being. Contextual demands and psychological pressures can cause burnout, which is a maladjustment characterized by feelings of exhaustion, cynicism, and inadequacy regarding school or sport activities (Fiorilli et al., 2017; Salmela‐Aro & Upadyaya, 2014; Salmela‐Aro, 2017; Sorkkila et al., 2020). Engagement is a positive state encompassing adolescents' capacity to plan, engage with, and assess their own involvement in schoolwork or sports and dream of future endeavors (Hastie et al., 2020; Wang, Degol, et al., 2019). Burnout and engagement are interrelated (Leiter & Maslach, 2017), and for DC athletes, they need to be assessed jointly in both school and sport contexts.

Previous research considering upper secondary DC athletes in sport schools (age 16–18 years) revealed that burnout and engagement develop consistently within school and sport (Ryba et al., 2021, 2017; Sorkkila et al., 2018). Furthermore, the findings provided preliminary evidence on cross‐contextual effects that align with the demands–resources model (Demerouti et al., 2001), suggesting that combining school and sport can either enhance engagement in both contexts or lead to burnout spilling over from one context to the other. Thus, examining engagement and burnout together could bridge a gap in previous research and provide insights into the mechanisms younger adolescent athletes might utilize to balance school and sport (Stambulova et al., 2015). Using quantitative data coming from lower secondary sport schools, this study examined the cross‐lagged reciprocal relationships between engagement and burnout in school and sport among Finnish adolescent athletes in Grades 8 and 9, controlling for gender.

### 1.1 School and sport burnout among adolescent athletes

School burnout may be a psychological syndrome or an emotional state arising from prolonged academic stress that transforms into burnout over time (Walburg, 2014). It is caused by a mismatch between a student's internal and external resources, school demands, and personal or environmental expectations of academic success (Salmela‐Aro et al., 2008). School burnout symptoms occur along a continuum from academic stress to major burnout, comprising three subdimensions—exhaustion, cynicism, and inadequacy (Salmela‐Aro et al., 2009). Exhaustion is a state of emotional fatigue due to increasing school demands, whereas cynicism involves a loss of interest and meaning in schoolwork. Inadequacy refers to diminished feelings of competence and achievement in tests, classes, and homework.

School burnout starts to develop among students at the end of primary education and increases steadily across lower secondary school and the transition to upper secondary education (ages 16–18 years) (Parviainen et al., 2021). A systematic review revealed that 60%–85% of adolescents with low/moderate initial burnout levels maintained stable or slightly increased burnout throughout lower secondary school (Vansoeterstede et al., 2023). Research suggests that DC athletes tend to mirror the general population in terms of mental health patterns (Knowles et al., 2021). Prolonged burnout can increase the risk of mental health problems, and research has shown that 17% of adolescent athletes aged 12–15 years report at least one mental health symptom (Brand et al., 2013). The prevalence of school burnout is roughly 30% among adolescent athletes aged 13–15 years (Saarinen et al., 2024). School burnout is particularly prevalent among nonathlete and athlete girls in lower secondary school (Saarinen et al., 2024; Salmela‐Aro et al., 2017). Burnout is also a context‐specific, age‐related phenomenon (Vansoeterstede et al., 2023), and pressure arising from two intertwined achievement domains can make DC athletes especially vulnerable to burnout in school and sports (Stambulova et al., 2023).

Sport burnout encompasses dimensions similar to school burnout, including physical and emotional exhaustion in response to intensified sport demands, cynical devaluation of sports, and a reduced sense of sport accomplishment and competence (Raedeke & Smith, 2001). Around 10% of adolescent DC athletes (aged 13–15 years) experience sport burnout in sport schools, with slightly higher rates among girls (Saarinen et al., 2024). Approximately 60% report low, stable levels of sport burnout over time in upper secondary sport schools (Sorkkila et al., 2020). There is a need to examine the development of sport burnout among lower secondary students and identify potential gendered differences in sport burnout symptoms (Isoard‐Gautheu et al., 2015). The three dimensions of burnout (i.e., cynicism, inadequacy, and exhaustion) generally remain stable and strongly correlated over time in school and sport contexts (Salmela‐Aro et al., 2017; Sorkkila et al., 2018). To ensure clarity, supported by previous research (Salmela‐Aro et al., 2009), we conceptualize burnout as a single universal construct composed of three separate dimensions that can be measured separately for school and sports.

### 1.2 School and sport engagement among adolescent athletes

Engagement is associated with success, positive emotions (i.e., enjoyment), and competence, facilitating the effective management of current academic and athletic pursuits and subsequent transitions in school and sports (Salmela‐Aro & Upadyaya, 2014; Scanlan et al., 2016). In contrast to burnout, engagement is a positive indicator of context‐specific well‐being (Hastie et al., 2020; Salmela‐Aro et al., 2021). Although sport engagement has attracted less interest than school burnout, they have been defined along behavioral, affective, and cognitive dimensions (Fredricks et al., 2004; Hastie et al., 2020). The behavioral dimension of engagement involves an individual's observable actions, such as focus and persistence regarding school or sports. The affective dimension reflects emotions regarding sports, school, and social interactions with peers, parents, and teachers. The behavioral and affective dimensions tend to fluctuate and explain students' motivation to be cognitively engaged in their academic work (Wang & Holcombe, 2010). The cognitive dimension involves relatively stable internal processes within an individual, including learning strategies and self‐regulation skills, as well as attitudes toward future learning in both academic and sport contexts (Hastie et al., 2020; Wang et al., 2019). The cognitive dimension is not easily measured through observation (Hastie et al., 2020); thus, in this study, we conceptualized sport and school engagement using the cognitive dimension.

Evidence is contradictory regarding the development of students' school engagement, with some studies suggesting a general decrease in cognitive engagement among the general student population aged 13–15 years (Wang & Eccles, 2012) and others reporting that subgroups of these students exhibit increasing cognitive engagement (Zhen et al., 2020). Adolescents' cognitive interest in sport participation tends to decrease, with a noticeable peak between 13 and 15 years of age (Blomqvist et al., 2023). Limited research suggests that female DC athletes may be particularly encouraged to prioritize cognitive effort in school and limit their investment in sports to minimize the uncertainty of a sporting career (Ronkainen et al., 2021; Saarinen et al., 2023). This challenge intensifies around age 15 when students face a critical transition to upper secondary school (Kuokkanen et al., 2024; Ryba et al., 2021).

### 1.3 Demands–resources model of the reciprocal relationship between burnout and engagement

Cross‐sectional prospective studies generally assume a simple, unidirectional relationship between engagement and burnout, hypothesizing that they are inversely related (Leiter & Maslach, 2017; Salanova et al., 2010). However, such simplification may be problematic, as theory and evidence indicate that the relationship between burnout and engagement may be complex and/or bidirectional, meaning that unidirectional tests can be inadequate and provide potentially misleading conclusions. Recent research suggests that burnout and engagement are distinct but related forms of well‐being (Molinari & Grazia, 2023; Salmela‐Aro, 2017) that are often combined as key factors in the demands–resources model (Demerouti et al., 2001).

The demands–resources model facilitates understanding of how the balance between adolescents' resources and demands affects their well‐being through two psychologically different processes (Demerouti et al., 2001; Salmela‐Aro & Upadyaya, 2014)—an effort‐driven process characterized by increasing demands (i.e., time spent in training or challenges in school) that result in stress and depletion of energy, subsequently leading to feelings of exhaustion and inadequacy and lower interest and belief in the meaningfulness of an activity, and second, a motivational process by which the availability of resources facilitates engagement and general life satisfaction (Demerouti et al., 2001).

Previous research supports the existence of an effort‐driven process leading to school burnout (Salmela‐Aro & Upadyaya, 2014), but sport demands may also trigger sport burnout among athletes (Martinent et al., 2021). Engagement and burnout are mutually causal and negatively associated within both school and sport domains (Guo et al., 2022; Widlund et al., 2023). Regarding the motivational process, personal attributes such as self‐efficacy and resources are likely to positively predict engagement and subsequent school burnout (Salmela‐Aro, 2017), although positive effects can spill over from other life areas to school (Salmela‐Aro & Upadyaya, 2014).

Adolescent DC athletes may be expected to assign equal amounts of time and energy to school and sports (Ryba et al., 2021, 2017). Regarding the motivational process, sport skills acquired through engagement may facilitate current academic success and future academic transitions (Kuokkanen et al., 2024; Storm & Eske, 2022), helping to control the development of burnout (Kuokkanen et al., 2022). These positive cross‐contextual effects may result from having sufficient DC resources (De Brandt et al., 2018). Regarding the effort‐driven process, adolescents occasionally need to lower their engagement in one domain to balance a misfit between multiple demands and individual and environmental resources (Eccles & Wigfield, 2002; Ryba et al., 2021; Stambulova et al., 2023). A lack of recovery due to high combined school and sport demands can trigger burnout in school, which might subsequently spill over to sports (Sorkkila et al., 2018).

### 1.4 The present study

Although evidence has shown that engagement and burnout are probably related, little is known about the direction of their effects and how they may translate between school and sport contexts. Thus, the aim of the current study was to examine the cross‐lagged reciprocal relationships between engagement and burnout in school and sports among Grade 8 and 9 Finnish adolescent DC athletes, controlling for gender. Supported by previous research on regular students and adolescent DC athletes in upper secondary sport schools (Blomqvist et al., 2023; Guo et al., 2022; Parviainen et al., 2021; Salmela‐Aro & Upadyaya, 2014; Sorkkila et al., 2018; Zhen et al., 2020) and guided by the demands–resources model (Demerouti et al., 2001), we hypothesized the following:

Engagement and burnout in both sports and school remain stable over time, exhibiting significant autoregressive effects.

There is a significant reciprocal relationship between school burnout and engagement, such that adolescent DC athletes with high burnout become less engaged over time, while those with low engagement become more burned out.

There is a significant reciprocal relationship between sport burnout and engagement, such that adolescent DC athletes with high burnout become less engaged over time, while those with low engagement become more burned out.

There are cross‐contextual effects of burnout and engagement across school and sport in both directions. As this among the first studies to examine these potential cross‐contextual effects, no specific hypothesis on the direction of these effects was proposed.

**Figure 1:**
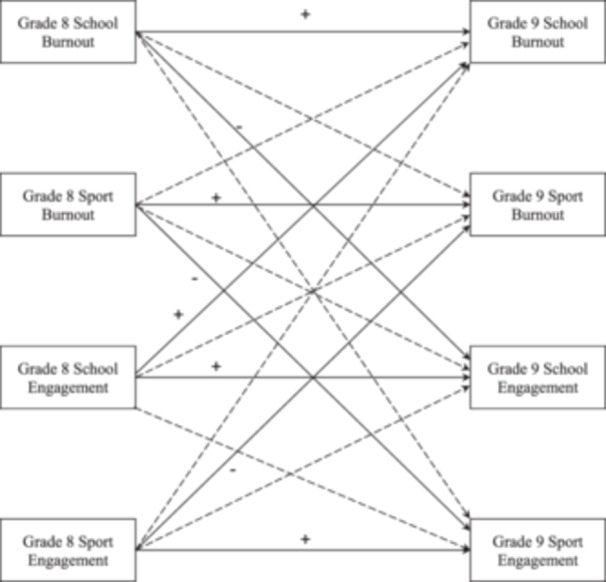
The cross‐lagged model of burnout and engagement in school and sport. *Note*. Solid lines indicate hypothesized paths and broken lines indicate hypothesized exploratory paths. + indicate a hypothesized significant regression path with a positive effect. ‐ indicate a hypothesized significant regression path with a negative effect.

Figure 1 presents the hypothesized within‐ and cross‐contextual paths from burnout and engagement in sport and school in Grade 8 to the corresponding constructs in Grade 9. Previous research suggests possible gender differences in the investigated variables; thus, we added gender to unravel the mean‐level differences between genders. Based on previous findings (Parviainen et al., 2021), we expected that girls would show higher levels of burnout and engagement in school than boys.

## 2 METHODS AND MATERIALS

### 2.1 Finnish lower secondary sport schools

Following an emerging European trend to establish specialized sports schools to facilitate adolescent athletes' pursuit of their academic and athletic ambitions, the Finnish Olympic Committee (2020) accredited 25 public mainstream lower secondary schools (ages 13–15 years) for this purpose. Each of the participating schools offered sport classes for approximately 25 DC athletes across different sports to train and study together on school premises as part of a lower secondary sport school pilot project (LSSSPP) during the 2017–2020 academic years. The overarching aim of the LSSSPP was to identify and develop local and national standards and guidelines for DC support and to find ways to strengthen collaboration between schools, local sport clubs, and academies. Therefore, these schools committed to providing 10 h of sport training during school hours in Grades 7–9 and teaching the essential DC skills for maintaining DCs in upper secondary school or vocational education (ages 16–18 years). These interesting forms of support meant that schools partly assumed the traditional role of sport clubs by developing athletes. The fact that adolescents were selected based on physical aptitude tests and grouped into specific sport classes within regular educational institutions that followed the general curriculum made this an interesting context and population to study.

### 2.2 Participants and procedure

We used a variable‐centered nonexperimental quantitative approach to test the four hypotheses because this approach is considered viable in the early phases of research aiming to empirically test the direction and strength of relationships among research variables (Cook & Cook, 2008). This study provides a foundation for subsequent quasi‐experimental and experimental studies to assess longitudinal cross‐lagged associations between engagement and burnout in school and sports.

This study is part of a larger longitudinal mixed‐methods research project Sport and Education for Life that examines the individual and environmental factors underpinning adolescent athletes' DC development and well‐being in Finland (Kuokkanen et al., 2022). The Board for Research Ethics at Abo Akademi University approved the research protocol before the recruitment of the study participants in 2017. Participants were recruited from 16 different lower secondary schools in various parts of Finland. All participating students and their parents gave their informed written consent to participate in the study. We followed the ethical guidelines of the Declaration of Helsinki throughout the study, including the participants' right to withdraw from the study without giving a reason and our own commitment to personal data protection. Longitudinal data were collected as part of the LSSSPP at four time points between the 2017 and 2020 academic years (Time 1, fall 2017; Time 2, spring 2018; Time 3, spring 2019; and Time 4, spring 2020). The measurement instruments employed with each wave of data collection differed slightly. For this particular study, we used quantitative data collected from adolescent athletes who were present at both T3 and T4. Participants responded anonymously to identical electronic questionnaires, which included items relevant to this study and other items relevant to the wider research project, during school hours under teachers' supervision in Grades 8 (age 14) and 9 (age 15). The sample comprised 165 adolescent DC athletes (48.9% girls), with a mean age of 14–15 years (M = 14.5, SD = 0.4) at T3. Data and data analysis codes are available from the first author upon reasonable request.

### 2.3 Measurements

#### 2.3.1 School burnout

We measured school burnout using the School Burnout Inventory (SBI; Salmela‐Aro et al., 2009). The instrument consists of nine items, of which four measure exhaustion at school (e.g., “I often sleep poorly because of matters related to my schoolwork”), three measure cynicism toward the meaning of school (e.g., “School doesn't interest me anymore”), and two measure feelings of inadequacy as a student (e.g., “I used to achieve more in school”). We merged the items to form a sum score, with Cronbach's alpha reliability coefficients of .90 for Grade 8 and .87 for Grade 9. All items were rated on a 5‐point Likert scale (1 = “completely disagree” to 5 = “completely agree”). Previous studies have verified the good validity and reliability of this school burnout scale (Salmela‐Aro et al., 2009).

#### 2.3.2 Sport burnout

We measured sport burnout using the Sport Burnout Inventory‐Dual Career Form (Sorkkila et al., 2020), which was adapted to the sport context from the School Burnout Inventory (Salmela‐Aro et al., 2009) and is an optimal tool for measuring sport burnout in a DC context. The scale comprises nine items, of which four measure sport‐related exhaustion (e.g., “I often sleep poorly because of matters related to my sport”), three measure cynicism toward the meaning of one's sport (e.g., “Sport doesn't interest me anymore”), and two measure feelings of inadequacy as an athlete (e.g., “I used to achieve more in my sport”). All items were rated on a 5‐point Likert scale (1 = “completely disagree” to 5 = “completely agree”). We created a mean score for the nine items to assess the overall level of sport burnout. The Cronbach's alpha reliability values for the scale were .94 for Grade 8 and .92 for Grade 9. Previous studies have verified the good validity and reliability of this sport burnout scale (Salmela‐Aro et al., 2009; Sorkkila et al., 2020).

#### 2.3.3 School engagement

We measured school engagement using the cognitive dimension of the Student Engagement Instrument Brief Version (Virtanen et al., 2018), including six items (e.g., “Learning is fun because I get better at something”) that we merged to form a mean score, with Cronbach's alpha values of .81 and .85 for Grades 8 and 9, respectively. All items were rated on a 5‐point Likert scale (1 = “completely disagree” to 5 = “completely agree”). These subscales have displayed good reliability and satisfactory validity in school contexts (Virtanen et al., 2018).

#### 2.3.4 Sport engagement

We measured sport engagement using the Sport Engagement Instrument (Kuokkanen et al., 2021), which was developed to examine sport engagement in school contexts, allowing for equal measures in both sport and school contexts. The cognitive dimension encompasses six items (e.g., “Sport is fun because I get better at something”) that we merged to form a mean score. The Cronbach's alpha values for the total scale were .88 and .89 for Grades 8 and 9, respectively. All items were rated on a 5‐point Likert scale (1 = “completely disagree” to 5 = “completely agree”). These subscales previously exhibited good reliability and satisfactory validity in sport contexts (Kuokkanen et al., 2021).

#### 2.3.5 Gender

Gender was added as a (0 = female, 1 = male) covariate in the analysis.

### 2.4 Data analysis

Data were fitted to a cross‐lagged path model using the Lavaan package in R, with missing data accounted for using full information maximum likelihood estimation (FIML). Specifically, school burnout, sport burnout, school engagement, and sport engagement in Grade 9 were regressed on the same constructs in Grade 8, controlling for gender. Model fit was assessed by comparing the cross‐lagged model with a model for autoregressive effects only (i.e., where each construct predicted only itself over time). A significant *χ* ^2^ change between the models and higher AIC and SABIC values in the stability‐only model indicated significant improvement of the model through the addition of cross‐lagged effects.

## 3 RESULTS

Missing survey data ranged from 0% to 12.7% but were deemed to be randomly missing according to a Little's MCAR test (*p* = .237). We constructed a path model to examine the cross‐lagged associations between adolescents' engagement and burnout in school and sports in Grades 8 and 9. Table 1 provides a full correlation table for all variables used in the present study. The cross‐lagged model showed good fit compared to the stability‐only model (*χ* ^2^ (12) = 30.69, *p* = .002, ΔAIC = 6.69, ΔSABIC = 7.40), predicting a modest portion of variance in all variables at follow‐up (Grade 9 school engagement *R* = .21, Grade 9 sport engagement *R* ^2^ = .35, Grade 9 school burnout *R* = .26, Grade 9 sport burnout *R* ^2^ = .30). Table 2 shows the parameter estimates for the cross‐lagged model alongside associated confidence intervals, standardized β regression coefficients, and Cohen's *f* ^*2*^ effect sizes for each parameter. School engagement, sport engagement, school burnout, and sport burnout all exhibited significant autoregressive effects, indicating a degree of stability over time. High levels of school engagement in Grade 8 were associated with low levels of engagement in sport in Grade 9, but not school‐ or sport‐related burnout. High levels of engagement in sport in Grade 8 predicted low levels of sport burnout in Grade 9 but not school‐related engagement or school burnout. Sport‐related burnout in Grade 8 predicted decreased levels of engagement in sport in Grade 9 but not school‐related engagement or burnout. School‐related burnout in Grade 8 was related to low levels of engagement in school in Grade 9 but not sport‐related engagement or burnout. Finally, we observed some significant effects of gender, with adolescent male DC athletes reporting lower levels of engagement in school and school burnout than girls in Grade 9.

**Table 1:** Correlations between the study variables.

	1	2	3	4	5	6	7	8	9
1. Gender	‐								
2. Cognitive Engagement Sport—Grade 8	.03	‐							
3. Cognitive Engagement Sport—Grade 9	.06	.50***	‐						
4. Cognitive Engagement School—Grade 8	−.12	.14	.01	‐					
5. Cognitive Engagement School—Grade 9	−.20*	.14	.22**	.37***	‐				
6. Sport Burnout—Grade 8	−.12	−.30***	−.33***	−.21**	−.17*	‐			
7. Sport Burnout—Grade 9	−.13	−.30***	−.48***	−.04	−.16	.52***	‐		
8. School Burnout—Grade 8	−.15	−.18*	−.14	−.21**	−‐.25**	.49***	.26**	‐	
9. School Burnout —Grade 9	−.27***	−.17*	−.28***	−.19*	−.38***	.32***	.44***	.44***	‐
Mean	‐	4.19	4.29	4.14	4.20	2.29	2.25	2.86	2.73
SD	‐	0.81	0.74	0.61	0.63	1.21	1.10	1.09	0.97
Cronbach's *α*	‐	.88	.85	.81	.89	.94	.92	.90	.87
McDonald's *ω*	‐	.89	.86	.82	.90	.94	.92	.90	.87
Missingness	0%	13%	7%	0%	1%	7%	13%	0%	1%

Abbreviation: SD, standard deviation.

* *p* < .05;

** *p* < .01;

*** *p* < .001.

**Table 2:** Parameter estimates for the cross‐lagged model of engagement and burnout in school and sports.

Parameter estimates	*B*	*SE*	*p*	Lower CI	Upper CI	*β*	*f* ^*2*^
Regression effects							
Sport Engagement Grade 8 → School Engagement Grade 9	0.06	0.07	.371	−0.07	0.19	.07	.005
School Engagement Grade 8 → School Engagement Grade 9	0.30	0.08	<.001	0.15	0.45	.29	.092
Sport Burnout Grade 8 → School Engagement Grade 9	−0.01	0.05	.802	−0.10	0.08	−.02	.000
School Burnout Grade 8 → School Engagement Grade 9	−0.11	0.05	.019	−0.20	‐0.02	−.19	.037
Gender → School Engagement Grade 9	−0.25	0.09	.005	−0.43	‐0.07	−.20	.042
Sport Engagement Grade 8 → Sport Engagement Grade 9	0.58	0.09	<.001	0.39	0.76	.51	.352
School Engagement Grade 8 → Sport Engagement Grade 9	−0.20	0.10	.050	−0.40	0.00	−.14	.020
Sport Burnout Grade 8 → Sport Engagement Grade 9	−0.13	0.06	.017	−0.24	−0.02	−.19	.037
School Burnout Grade 8 → Sport Engagement Grade 9	−0.03	0.06	.603	−0.16	0.09	−.04	.002
Gender → Sport Engagement Grade 9	−0.06	0.12	.586	−0.30	0.17	−.04	.002
Sport Engagement Grade 8 → Sport Burnout Grade 9	−0.30	0.13	.019	−0.54	−0.05	−.20	.042
School Engagement Grade 8 → Sport Burnout Grade 9	0.14	0.14	.297	−0.13	0.41	.08	.006
Sport Burnout Grade 8 → Sport Burnout Grade 9	0.41	0.08	<.001	0.26	0.56	.45	.254
School Burnout Grade 8 → Sport Burnout Grade 9	0.03	0.09	.739	−0.14	0.20	.03	.001
Gender → Sport Burnout Grade 9	−0.09	0.16	.559	−0.41	0.22	−.04	.002
Sport Engagement Grade 8 → School Burnout Grade 9	−0.08	0.10	.409	−0.27	0.11	−.06	.004
School Engagement Grade 8 → School Burnout Grade 9	−0.18	0.11	.112	−0.40	0.04	−.11	.012
Sport Burnout → School Burnout Grade 9	0.07	0.07	.265	−0.06	0.20	.09	.008
School Burnout Grade 8 → School Burnout Grade 9	0.29	0.07	<.001	0.15	0.43	.33	.122
Gender → School Burnout Grade 9	−0.42	0.13	.001	−0.68	‐0.16	−.22	.051
Covariances							
Sport Engagement Grade 8 ↔ School Engagement Grade 8	0.06	0.04	.079	−0.01	0.13	.14	.020
Sport Engagement Grade 8 ↔ Sport Burnout Grade 8	−0.26	0.07	<.001	−0.41	−0.12	−.30	.099
Sport Engagement Grade 8 ↔ School Burnout Grade 8	−0.14	0.07	.032	−0.27	−0.01	−.17	.030
Sport Engagement Grade 8 ↔ Gender	0.02	0.03	.551	−0.04	0.08	.05	.003
School Engagement Grade 8 ↔ Sport Burnout Grade 8	−0.16	0.06	.008	−0.27	−0.04	−.21	.046
School Engagement Grade 8 ↔ School Burnout Grade 8	−0.14	0.05	.007	−0.24	−0.04	−.21	.046
School Engagement Grade 8 ↔ Gender	−0.04	0.02	.116	−0.08	0.01	−.12	.015
Sport Burnout Grade 8 ↔ School Burnout Grade 8	0.65	0.12	<.001	0.42	0.88	.49	.316
Sport Burnout Grade 8 ↔ Gender	−0.08	0.05	.105	−0.17	0.02	−.13	.017
School Burnout Grade 8 ↔ Gender	−0.08	0.04	.057	−0.17	0.00	−.15	.023

Abbreviation: CI, confidence interval; SE, standard error.

## 4 DISCUSSION

The aim of the current study was to examine the cross‐lagged reciprocal relationships between engagement and burnout in school and sports among a sample of Grade 8 and 9 Finnish adolescent DC athletes, controlling for gender. The longitudinal nature of the data (two waves 1 year apart) provides a valuable basis for understanding the relationships between burnout and engagement among a constantly growing subgroup of students who face increasing school and sport demands at a sensitive developmental stage before transferring to upper secondary education. Aligning with the demands–resources model (Demerouti et al., 2001), the results showed that adolescent DC athletes developed relatively stable engagement and burnout within school and sport. They also revealed intriguing spillover effects between school and sport engagement, suggesting the existence of cross‐contextual associations that have rarely been examined in previous studies.

In line with H1, engagement in sport and school remained stable between Grades 8 and 9, with sport engagement showing a stronger positive autoregressive path than school engagement. This contrasts with the general adolescent athlete population in sport clubs, where engagement tends to decline around the age of 15 (Blomqvist et al., 2023). Potentially, DC athletes in specialized sport schools may benefit from combined training during school hours and evening sessions at their sport clubs, since increased training hours generally boost cognitive and behavioral sport engagement (Martinent et al., 2021). Adolescent athletes train and study together with like‐minded peers in sport schools, which may strengthen their general interest and provide extended support for sports, potentially facilitating current and future engagement.

Another explanation is that little extra cognitive effort is required to maintain academic success, since adolescent athletes often achieve higher grades than their nonathlete peers (Storm & Eske, 2022). Simultaneously, they recognize the need to start investing more heavily in training during the last year of compulsory education (Grade 9) to meet intensifying sport demands and acquire sufficient athletic (e.g., competition success) merits for transitioning to specialized upper secondary sport schools (Kuokkanen et al., 2024). This is also an efficient strategy to maintain DCs in the long term (Ryba et al., 2021, 2017).

Confirming previous findings (Guo et al., 2022; Sorkkila et al., 2018), the results related to H1 showed that burnout in Grade 8 was modestly and positively correlated with burnout in Grade 9. The relationship was slightly stronger in sport than in school, perhaps because, while adolescents experience occasional extra exam stress, school demands remain consistent, whereas time spent on training and achieving personal success in sport increases during the final year of lower secondary education (Kuokkanen et al., 2024; Stambulova et al., 2015). Practitioners working with DC athletes should note that this result may also indicate that the adolescents had developed sufficient DC competencies and skills (e.g., time management skills and resilience; De Brandt et al., 2018) to increase their involvement in sports while maintaining low burnout levels over time.

The results supported the first parts of H2 and H3, indicating a weak negative relationship between high levels of burnout in Grade 8 and low levels of engagement in school and sport in Grade 9. These findings suggests that adolescent athletes can cope with a misfit between multiple demands and individual and environmental resources that causes initial burnout by lowering their engagement in either school or sports rather than developing burnout (Eccles & Wigfield, 2002; Stambulova et al., 2024). For adolescent athletes, reducing engagement in one domain may be a functional short‐term coping strategy to prevent burnout (Kuokkanen et al., 2022). However, prolonged reduced engagement in either domain could undermine their future capacities to sustain DCs (Ryba et al., 2021).

The results partly supported the latter parts of H2 and H3, with high sport engagement in Grade 8 associated with low levels of sport burnout in Grade 9. Based on the demands–resources model (Demerouti et al., 2001; Salmela‐Aro & Upadyaya, 2014), sport engagement can reduce burnout by contributing to a sense of competence and satisfaction that balance the increasing demands placed on adolescent athletes. The finding has value for school practitioners and educational policy, suggesting that extended sport training during school hours can contribute to athletic development and facilitate adolescent athletes' well‐being. Engaging in schoolwork can also be rewarding and enjoyable; however, adolescent DC athletes often view it primarily as a necessity (Ryba et al., 2017). Consequently, this may explain why a similar positive relationship between engagement and burnout observed for sport did not apply to school in this study.

Regarding H4, the results revealed that high school engagement in Grade 8 was associated with low sport engagement in Grade 9, but the relationship did not hold for sport engagement and school engagement. It may be that high levels of school engagement consume significant cognitive resources, resulting in fewer resources for sport engagement (Salmela‐Aro & Upadyaya, 2014). Alternatively, adolescent DC athletes' high self‐imposed demands in school may restrict the time and energy available for engaging in sports (Ryba et al., 2017). In contrast to older DC athletes (16 + ) navigating increasing DC demands by allowing school burnout spill over into sports (Sorkkila et al., 2018), the results revealed no such cross‐domain burnout associations. This suggests that burnout is specific to the domain in which a demands–resources imbalance occurs. Overall, the existent and nonexistent findings indicate that reducing sport engagement may be the primary mechanism for maintaining adolescent DC athletes' high school engagement.

Regarding mean‐level differences between genders, female DC athletes reported higher levels of school engagement and school burnout than boys, which aligns with previous results regarding regular students in lower secondary school (Salmela‐Aro et al., 2017). Based on previous DC research (Ryba et al., 2021; Saarinen et al., 2023), the gender difference in school engagement may have stemmed from girls experiencing social pressure to achieve academic success rather than a lack of study resources. In particular, high cognitive engagement requires considerable effort (Salmela‐Aro & Upadyaya, 2014), and potential perfectionistic tendencies toward school may contribute to higher school burnout rates among girls (Ronkainen et al., 2021).

## 5 LIMITATIONS AND FUTURE DIRECTIONS

First, regarding the limitations of this study, the researchers employed a two‐wave design and obtained inherently valuable longitudinal data. However, future research should incorporate additional measurement points to examine how burnout and engagement evolve within and across school and sport in Grades 7–9 and in upper secondary school. Furthermore, it could be interesting to examine whether the relationships between the constructs and their magnitudes differ according to whether DC athletes choose a regular or sport‐oriented track in upper secondary school (aged 16–18 years), when school and sport demands intensify and when they transition from adolescence into adulthood.

Second, the small study sample may have affected the accuracy of the estimates. Replicating these findings with a larger sample would allow for more complex analyses and more precise estimates, such as decomposing burnout into its three dimensions (exhaustion, cynicism, and inadequacy) and assessing their relationships with cognitive engagement. Future research should also consider the behavioral and affective components of engagement to provide a more comprehensive understanding of how these dimensions interact within and across school and sport. A larger sample size would also enable using latent transition analysis, potentially providing valuable insights into athletes' well‐being by profiling DC athletes based on their engagement and burnout levels in both school and sport, and then examining changes in profiles over time to identify athletes at risk and those who excel in balancing the two domains.

## 6 CONCLUSION AND PRACTICAL IMPLICATIONS

The findings of the current study have theoretical and practical implications for educators, coaches, and researchers working with adolescent DC athletes. The demands–resources model provides a viable framework for further assessing DC athletes’ well‐being. The findings regarding the development of burnout and engagement within school and sport can be generalized to other sport school settings, as they align with previous research on older DC athletes (Ryba et al., 2021; Sorkkila et al., 2018), highlighting that school and sport are distinct but intertwined domains, each with its own set of demands and resources. Regarding the demands‐resource model (Salmela‐Aro & Upadyaya, 2014), the results support the existence of an effort‐driven (i.e., high burnout predicting less engagement) process in school and sport, and they provide novel insights into the existence of a potential motivational process in sports (i.e., high sport engagement resulting in less sport burnout over time). This study also provides preliminary evidence that adolescent DC athletes can maintain high school engagement by lowering their levels of sport engagement (e.g., through an effort‐driven process). There is a need to verify this and other negative and positive cross‐domain mechanisms that adolescents might use to balance their DCs in future research. For instance, sport engagement could potentially lead to increased school engagement in environments where school and sport are more closely integrated.

It is crucial for coaches, teachers, and parents to recognize that engagement and burnout appear to be consistent and context dependent. Regular individual discussions could be key to identifying obstacles to school or sport engagement, mitigating strain, and preventing burnout. It is essential to recognize girls' high self‐imposed success expectations in school. Coaches could help by reducing sporting demands during peak school periods to maintain their students' involvement in sports, and teachers could foster self‐care through regular discussions. Coaches could challenge boys with low levels of burnout to invest more time and energy in their sporting development, and parents could monitor daily fatigue to help adolescents balance their combined school and sport workloads.

Furthermore, developing interventions and policies to proactively address imbalances between demands and resources might help DC athletes use more efficient strategies beyond merely lowering engagement or developing symptoms of burnout to maintain a balance between school and sport. A potential early intervention might be to teach adolescent athletes DC competencies, including DC management strategies, career planning skills, emotional awareness, social intelligence, and adaptability (De Brandt et al., 2018). Schools and sport clubs could collaborate to teach these skills, and parents could ensure their daily application. These skills become particularly important for maintaining well‐being after the transition to upper secondary education, when the demands of both school and sport intensify (Ryba et al., 2021).

## AUTHOR CONTRIBUTIONS

**Joni Kuokkanen**: Conceptualization; data curation; methodology; investigation; funding acquisition; writing (original draft). **Milla Saarinen**: Conceptualization; investigation; validation; writing (original draft). **Daniel J. Phipps**: Conceptualization; formal analysis; methodology; visualization; investigation; writing (original draft). **Johan Korhonen**: Conceptualization; methodology; writing (review and editing); supervision. **Jan‐Erik Romar**: Conceptualization; funding acquisition; investigation; writing (review and editing); funding acquisition; supervision.

## CONFLICT OF INTEREST STATEMENT

The authors have no conflicts of interest to declare.

## ETHICS STATEMENT

The Board for Research Ethics at Abo Akademi University approved all procedures before recruiting the study participants in 2017.

## Data Availability

The data that support the findings of this study are available from the corresponding author upon reasonable request.
